# 17*β*-Estradiol Attenuates LPS-Induced Macrophage Inflammation In Vitro and Sepsis-Induced Vascular Inflammation In Vivo by Upregulating miR-29a-5p Expression

**DOI:** 10.1155/2021/9921897

**Published:** 2021-06-09

**Authors:** Man-li Zhang, Hui Chen, Zhan Yang, Man-na Zhang, Xia Wang, Kun Zhao, Xuan Li, Nan Xiu, Fei Tong, Ya-xuan Wang

**Affiliations:** ^1^Department of Critical Care Medicine, The Second Hospital of Hebei Medical University, 215 Heping West Road, Shijiazhuang, Hebei 050000, China; ^2^Department of Urology, The Second Hospital of Hebei Medical University, 215 Heping West Road, Shijiazhuang, Hebei 050000, China; ^3^Department of Talent and Academic Exchange Center, The Second Hospital of Hebei Medical University, 215 Heping West Road, Shijiazhuang, Hebei 050000, China; ^4^Department of Clinical Laboratory, The Second Hospital of Hebei Medical University, 215 Heping West Road, Shijiazhuang, Hebei 050000, China

## Abstract

Excessive release of cytokines such as IL-1*β* and other inflammatory mediators synthesized and secreted by macrophages is the fundamental link of uncontrolled inflammatory response in sepsis. 17*β*-Estradiol (E2) plays anti-inflammatory and vascular protective effects by regulating leukocyte infiltration and the expression of chemokines or cytokines induced by injury. However, the role of E2 in the inflammatory response of macrophages in sepsis and its mechanism are still not fully understood. In the present study, we show that E2 alleviates vascular inflammation in sepsis mice induced by cecal ligation puncture (CLP). E2 significantly decreases RAW 264.7 cell inflammation response by downregulating the expression of NLRP3. Furthermore, we found that miR-29a-5p was significantly downregulated in LPS-treated macrophages. Treating RAW 264.7 cells with E2 markedly upregulated the miR-29a-5p expression level. More importantly, we demonstrated that miR-29a-5p repressed NLRP3 expression by directly targeting its 3′-UTR. Loss- and gain-of-function experiments revealed that transfection of the miR-29a-5p mimic abrogates LPS-induced macrophage inflammation. Moreover, depletion of miR-29a-5p by its inhibitor largely promotes LPS-induced macrophage inflammation. In summary, miR-29a-5p upregulation induced by E2 alleviated RAW 264.7 cell inflammation response by aggravating miR-29a-5p repression of NLRP3 expression. E2 exerts significant anti-inflammatory efficacy in macrophages by regulating the miR-29a-5p/NLRP3 axis. Targeting miR-29a-5p may be a novel therapeutic strategy to suppress sepsis-induced vascular inflammation.

## 1. Introduction

Sepsis is a systemic inflammatory response syndrome caused by the host's dysregulated response to infection [1]. The pathophysiological mechanism of sepsis is very complex, and the current consensus is to activate both the proinflammatory and anti-inflammatory responses [2]. In the early stage of sepsis, macrophages promote host defense by eliminating invading pathogens or damaged tissues and releasing abundant amounts of proinflammatory cytokines, such as TNF-*α*, IL-1*β*, and IL-6 [3]. However, macrophages may be excessively activated in the early stage and produce excessive proinflammatory cytokines, which not only leads to microvascular injury of endothelial cells but also activates coagulation and complement cascade reaction, further aggravating vascular injury [4, 5]. These events are related to the clinical symptoms and signs of sepsis and the progression from sepsis to septic shock. Therefore, inhibiting macrophages from producing excessive proinflammatory cytokines is of great significance to reduce sepsis-induced vascular injury.

The NLR family pyrin domain-containing 3 (NLRP3) inflammasome, which is a kind of polymer protein complex, can finely regulate the activation of caspase-1 and mediate the production and secretion of proinflammatory cytokines IL-1*β* and IL-18. The NLRP3 inflammasome is involved in many inflammatory diseases, including sepsis. NLRP3 inflammasome inhibition attenuates sepsis-induced multiorgan injury in cecal ligation puncture (CLP) [6]. Inhibition of TLR4 and NLRP3 signaling pathways can protect sepsis-induced myocardial inhibition [7]. In sepsis mice and LPS-induced macrophages, autophagy-induced inactivation of the NLRP3 inflammasome can reduce the release of proinflammatory cytokines [8]. Therefore, it is important to understand how NLRP3 participates in the inflammatory response of macrophages in sepsis.

MicroRNAs (miRNAs) do not encode proteins but regulate gene expression. A variety of miRNAs have been confirmed to be involved in the development of sepsis. miR-146a blocks the activation of NF-*κ*B by targeting IRAK1 and TRAF6 expression and reduces sepsis-induced cardiac dysfunction, inflammatory cell infiltration, and inflammatory cytokine production [9]. miR-21 mediates apoptosis and septic shock by promoting the activation of the NLRP3 inflammasome in mouse and human bone marrow cells [10]. miR-29a can protect cells from damage by targeting JAK-STAT3 to inhibit IL-10-induced cytokine release during sepsis [11]. Noncoding RNA CRNDE protects the myocardium from sepsis-induced cardiomyocyte apoptosis and oxidative damage by inhibiting the transcriptional regulation of miR-29a on SIRT1 [12]. Gracillin attenuates mouse heart injury by improving miR-29a and inhibiting LPS-induced apoptosis and inflammation [13]. However, it is unclear whether and how miR-29a-5p mediates the regulation of macrophage inflammation.

As a major female hormone, estrogen has been proved to have anti-inflammatory, antioxidant, and organ-protective effects. Estrogen has been used as a therapeutic drug in many diseases, such as inflammatory bowel disease [14], acute spinal cord injury [15], neurovascular disease [16], and obesity [17]. Our previous study found that 17*β*-estradiol (E2) suppressed proinflammatory gene expression through promoting the interaction of estrogen receptor (ER) *α* with NF-*κ*B p50 and decreasing high glucose-induced interaction of KLF5 with NF-*κ*B p50 in vascular smooth muscle cells [18]. Sepsis is a systemic inflammatory response. Previous studies have shown the protective effect of estrogen in sepsis. Estrogen protects sepsis-induced liver injury by inhibiting ROS-mediated NLRP3 activation and mitochondrial dysfunction [19]. E2 can boost ROS production and RUBICON expression to further promote macrophage LC3B-associated phagocytosis [20]. E2 inhibited the nuclear translocation of RelB, which is a member of the noncanonical pathway of NF-*κ*B and contributes to macrophage polarization to change the intensity of trained immunity in sepsis [21]. Although we have a new understanding of the sepsis protective mechanism of E2, whether and how E2 inhibits sepsis-induced macrophage inflammatory response mediated by miR-29a-5p remains unclear.

In this study, we used mouse models of CLP and cultured macrophages to investigate whether and how E2 plays a protective role on CLP-induced vascular inflammation by regulating the expression of miR-29a-5p.

## 2. Materials and Methods

### 2.1. Animal Models

All animal studies were approved by the Institutional Animal Care Committee of Hebei Medical University, and all efforts were made to minimize suffering. 6-8-week-old male wild-type C57BL/6 mice were fed adaptively for a week. Capsule osmotic pumps (ALZET® Osmotic Pump 2004, ALZET, USA) containing E2 (30 *μ*g/kg/d, Merck, Germany) were implanted in the CLP+E2 group 5 days before CLP, continuing for 6 days thereafter, whereas the NC group and CLP group implanted capsule osmotic pumps without E2. The mice were anesthetized by inhalation with 1.5% isoflurane, fixed in the prone position, prepared to be skinned, disinfected, and covered with a sterile hole towel. Open the disinfection instrument package, and cut a 1 cm transverse incision on the back of the mice. Subsequently, a subcutaneous tunnel was prepared; insert the capsule osmotic pumps with the flow regulator facing the head side, and sew the subcutaneous tissue layer by layer to continuously sew the skin. After the operation, the mice were placed on the sterile heat pad and put into the animal room for single cage feeding. Five days later, the mice were subjected to CLP operation. CLP was performed as described previously [22]. Briefly, the mice were anesthetized with 1.5% isoflurane. A 1 cm midline incision was made, and the cecum was carefully exposed to avoid damage to the blood vessels. The cecum was then tightly ligated with a 3/0 silk suture at the middle and punctured twice using a 21-gauge needle at about 0.5 cm from its distal end. A small amount of stool was extruded to ensure patency of the puncture sites. The cecum was placed back into its normal intra-abdominal position, and the abdomen was closed in two layers. Mice in the sham group (NC group) underwent exactly the same procedures without CLP. All animals received a subcutaneous injection of 1 ml of 37°C normal saline for fluid resuscitation immediately after surgery. The tramadol hydrochloride analgesic (10 ml/kg, Germany) was administered by hypodermic injection every 12 h for 24 h. Blood samples were collected before surgery (0 h) and at 6, 12, and 24 h after surgery. After 24 h of operation, all mice were anesthetized and perfused with cold 0.9% NaCl, and the thoracic and abdominal aorta was collected for analysis of RNA, protein, and histology.

### 2.2. Cell Culture and Treatment

Murine macrophage cells (RAW 264.7) were routinely cultured in the RPMI 1640 medium containing 10% FBS at 37°C in a 5% CO_2_ incubator. Cells were treated for different times or concentrations with LPS in a 2% FBS-supplemented medium. Macrophages were also stimulated with different times or concentrations of E2 plus LPS (1 *μ*g/ml). 293A cells were maintained in high-glucose Dulbecco's modified Eagle's medium (DMEM, Gibco Life Technologies, Rockville, MD) supplemented with 10% FBS.

### 2.3. Transfection and Plasmid Constructs

All cells were transfected using Lipofectamine 2000 (Invitrogen) according to the manufacturer's protocol. The miR-29a-5p mimic/inhibitor and their respective controls were purchased from GenePharma Co., Ltd. Twenty hours after transfection, the RAW 264.7 cells were treated with 10% FBS. The cells were then harvested and lysed for qRT-PCR and Western blotting analysis. Restriction enzyme digestion and one-step cloning (ClonExpress II One-Step Cloning Kit, C112-02; Vazyme Biotech Co., Ltd.) were used to construct the luciferase reporter plasmids. The fragment of the mouse NLRP3 3′-UTR containing the miR-29a-5p binding site and its mutant sequences (Supplemental Table S1) were synthesized, respectively, and then were inserted into the pmirGLO Dual-Luciferase miRNA Target Expression Vector (GenePharma, Suzhou) digested by Xho1 and Sal1.

### 2.4. Isolation of RNA and Real-Time PCR

Total RNA was extracted by using the QIAzol Lysis Reagent according to the manufacturer's protocol. A biospectrometer (Thermo NanoDrop 2000) was used to determine the quality of the RNA. For microRNA analysis, the miScript II RT kit (QIAGEN GmbH) was used for reverse transcription, and the miScript SYBR Green PCR kit was used for qRT-PCR with specific primers for microRNAs. RNU6b (U6) was used as an internal control. For mRNA analysis, total RNA was reverse-transcribed to the first-strand cDNA with the M-MLV First-Strand Kit (Life Technologies). And real-time PCR analysis was done with the ABI 7500 FAST system, using the Platinum SYBR Green qPCR SuperMix UDG Kit (Invitrogen), according to the manufacturer's instructions. The relative amount of gene expression was normalized with GAPDH. All PCRs were performed in triplicate. The relative amount of transcripts was calculated using the 2^−*ΔΔ*Ct^ formula as previously described [23]. The primer sequences used throughout this study are listed in Supplemental Table S2.

### 2.5. Western Blotting Analysis

Proteins from cultured cells or vascular tissues were prepared with the lysis buffer (1% Triton X-100, 150 mM NaCl, 10 mM Tris-HCl, pH 7.4, 1 mM EDTA, 1 mM EGTA, pH 8.0, 0.2 mM Na_3_VO_4_, 0.2 mM phenylmethylsulfonyl fluoride, and 0.5% NP-40). Equal amounts of protein were separated by SDS-PAGE and transferred onto a PVDF membrane (Millipore). Membranes were blocked with 5% milk in TTBS for 2 h at 37°C and incubated overnight at 4°C with the following primary antibodies: anti-IL-1*β* (1 : 1000, ab9722, Abcam), anti-NLRP3 (1 : 1000, ab263899, Abcam), and anti-*β*-actin (1 : 1000, sc-47778, Santa Cruz Biotechnology). Membranes were washed and incubated with appropriate secondary antibodies for 1 h at room temperature. Protein bands were treated with the Immobilon™ Western (Millipore) and detected by ECL (enhanced chemiluminescence) Fuazon Fx (Vilber Lourmat).

### 2.6. Immunofluorescence Staining

Immunofluorescence staining was performed with 4 *μ*m paraffin cross-sections from the vascular tissues, deparaffinized with xylene and rehydrated. Nonspecific sites were blocked by incubation in 10% normal goat serum (710027, KPL, USA) for 1 h. Then, the sections or cells were incubated with primary antibodies at 4°C overnight. The primary antibodies were anti-MAC-2 (60207-1, Proteintech) and anti-SM22-*α* (ab14106, Abcam). Secondary antibodies were the rhodamine-labeled antibody to rabbit IgG (031506, KPL, USA) and fluorescein-labeled antibody to mouse IgG (021815, KPL, USA) or the rhodamine-labeled antibody to mouse IgG (031806, KPL, USA) and fluorescein-labeled antibody to rabbit IgG (021516, KPL, USA). Nuclei were stained with DAPI (157574, MB Biomedical) in each experiment. Images were captured by confocal microscopy (DM6000 CFS, Leica) and processed by LAS AF software.

### 2.7. Target Prediction

Potential target microRNAs of NLRP3 were identified with the following prediction algorithms: miRanda (http://www.microrna.org) and RNAhybrid (http://bibiserv.techfak.uni-bielefeld.de/rnahybrid/submission.html) [24, 25].

### 2.8. Luciferase Assays

293A cells were seeded into a 24-well plate, and the miR-29a-5p mimic (or mimic-ctl) was cotransfected with the NLRP3 reporter construct (wild type or mutant) or the empty vector. Twenty-four hours after transfection, cells were harvested in the lysis buffer. The Dual-Glo Luciferase Assay System (Promega) was used to detect luciferase activity according to the manufacturer's protocol. Firefly luciferase (FLuc) activity was measured and normalized against Renilla luciferase (RLuc) activity.

### 2.9. ELISA

The concentration of IL-1*β* in plasma was detected using a commercial ELISA kit (Proteintech, China) according to the manufacturer's instructions. The absorbance at 450 nm was read with a microtiter plate reader (SPECTRAFluor Plus, Tecan).

### 2.10. Statistical Analysis

All of the data are presented as the mean ± SEM. Differences between the two groups were analyzed by Student's *t*-test or by analysis of variance (ANOVA). For all analyses, a value of *P* < 0.05 was considered significant. All of the presented data were repeated in at least three independent experiments.

## 3. Results

### 3.1. E2 Alleviates Vascular Inflammation in Sepsis Mice Induced by CLP

**Figure 1:**
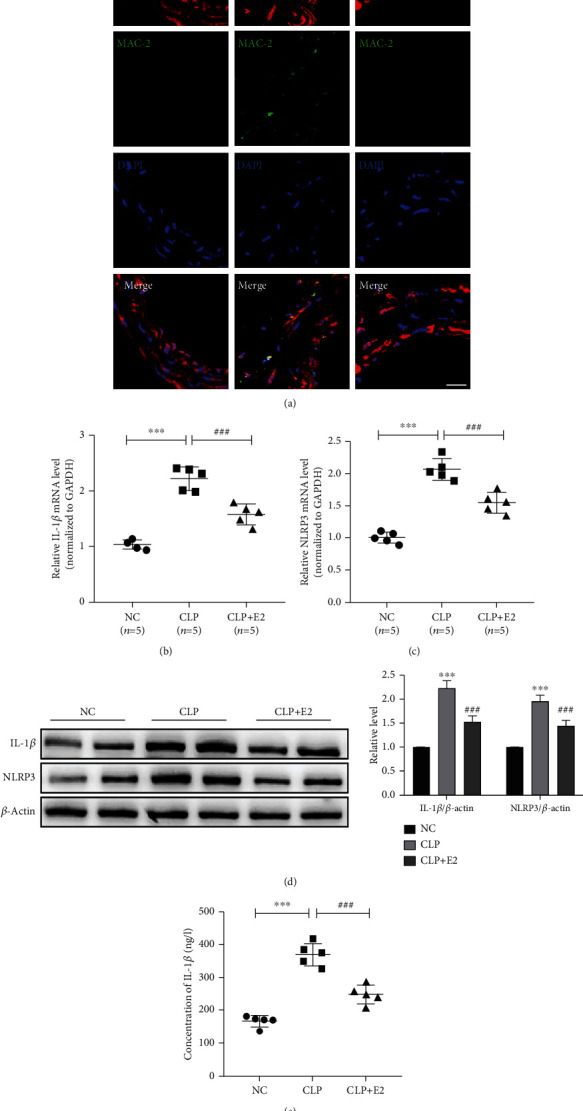
E2 alleviates vascular inflammation in sepsis mice induced by CLP. (a) Immunofluorescence staining of SM22-*α*, MAC-2, and the nucleus on the vascular tissues of the control (NC), CLP, and CLP+E2 groups. Red and green stainings indicate SM22-*α* and MAC-2, respectively. Blue staining indicates the nucleus. Scale bars = 10 *μ*m. (b, c) IL-1*β* and NLRP3 expression in NC-, CLP-, and CLP+E2-treated vascular tissues was detected by qRT-PCR 24 h after CLP. Relative expression of IL-1*β* and NLRP3 mRNA was presented after normalizing to GAPDH. *n* = 5 in each group. ^∗∗∗^*P* < 0.001 vs. the NC group. ^###^*P* < 0.001 vs. the CLP group. (d) IL-1*β* and NLRP3 expression in NC-, CLP-, and CLP+E2-treated vascular tissues was determined by Western blotting 24 h after CLP. Left panel: representative blot from five independent experiments. Right panel: quantitation of IL-1*β* and NLRP3 normalized to *β*-actin. ^∗∗∗^*P* < 0.001 vs. the NC group. ^###^*P* < 0.001 vs. the CLP group. (e) IL-1*β* in plasma was measured by ELISA. *n* = 5 in each group. ^∗∗∗^*P* < 0.001 vs. the NC group. ^###^*P* < 0.001 vs. the CLP group.

Because excessive inflammatory response caused by macrophage infiltration is the premise of vascular injury caused by sepsis, we examined the macrophage contents in vascular tissues of sepsis mice by immunofluorescence staining. The results showed that the macrophage numbers in the vessels (MAC-2-positive cells) of sepsis mice were readily detectable, whereas they were barely observed in the vessels of the E2-treated group, similar to the control group (Figure 1(a)). These observations suggest that sepsis can cause macrophage infiltration, and macrophages are likely the primary cellular source of vascular inflammation in sepsis mice. Previous studies have shown that estrogen has anti-inflammatory and organ-protective effects in sepsis; we first tested inflammatory cytokine expression in vascular tissues. As shown in Figures 1(b) and 1(d), compared with the NC group, qRT-PCR and Western blotting results revealed that expression levels of proinflammatory cytokine IL-1*β* mRNA and protein significantly increased in the vascular tissues of the CLP group, whereas treatment with E2 obviously decreased vascular inflammation response compared with the CLP group. Subsequently, we detected whether NLRP3 is involved in the upregulation of inflammatory cytokine expression in the vascular tissues. The expression level of NLRP3 mRNA remarkably increased in the vascular tissues of the CLP group. Notably, CLP-induced upregulation of NLRP3 was reversed by E2 treatment (Figure 1(c)). A similar result was obtained by Western blotting analysis of NLRP3, consistent with its mRNA expression (Figure 1(d)). We also further confirmed that treatment with E2 decreased the concentration of IL-1*β* in plasma induced by CLP (Figure 1(e) and Figure S1). These data indicate that E2-suppressed sepsis-induced proinflammatory cytokine production is correlated with NLRP3.

### 3.2. LPS Induces the Expression of NLRP3 and IL-1*β* in Cultured RAW 264.7 Cells

**Figure 2:**
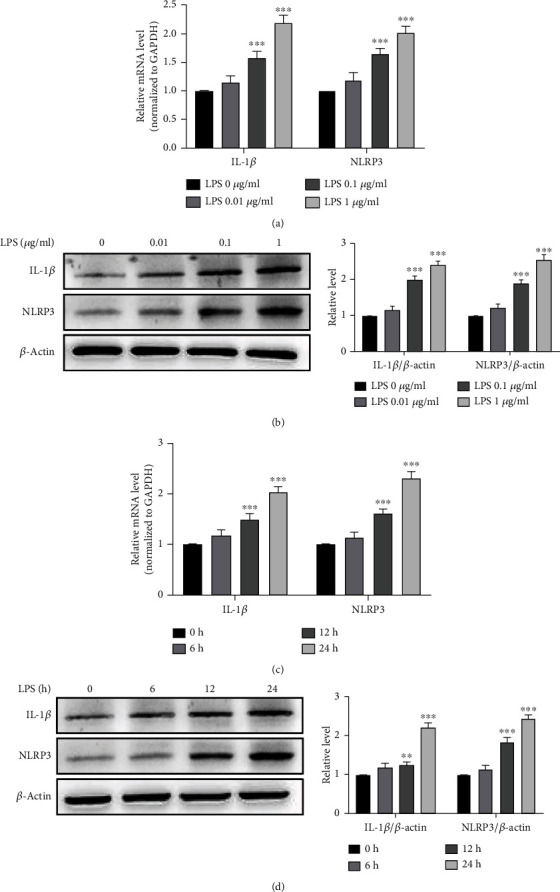
LPS induces the expression of NLRP3 and IL-1*β* in cultured RAW 264.7 cells. (a, b) RAW 264.7 cells were treated with the indicated concentrations of LPS for 24 h. qRT-PCR (a) and Western blotting (b) detected the mRNA and protein expression of IL-1*β* and NLRP3. Relative expression of IL-1*β* and NLRP3 mRNA was presented after normalizing to GAPDH (mean ± SEM; *n* = 3). ^∗∗∗^*P* < 0.001 vs. the LPS 0 *μ*g/ml group. (c, d) RAW 264.7 cells were treated with 1 *μ*g/ml LPS for the indicated times, and the mRNA and protein expression of IL-1*β* and NLRP3 was detected by qRT-PCR (c) and Western blotting (d). Relative expression of IL-1*β* and NLRP3 mRNA was presented after normalizing to GAPDH (mean ± SEM; *n* = 3). ^∗∗^*P* < 0.01 and ^∗∗∗^*P* < 0.001 vs. the 0 h group, respectively.

To explore the effect of NLRP3 on the sepsis-induced inflammatory response, we detected NLRP3 expression in LPS-stimulated macrophages. As shown in Figures 2(a) and 2(c), the addition of LPS to the RAW 264.7 cells led to dose- and time-dependent increases in NLRP3 and IL-1*β* mRNA levels, with mRNA levels of NLRP3 or IL-1*β* being more than 2-fold upregulated in 1 *μ*g/ml LPS-treated RAW 264.7 cells. Simultaneously, LPS also increased the level of NLRP3 and IL-1*β* proteins in a dose- and time-dependent manner (Figures 2(b) and 2(d)), reaching its maximal effects at 1 *μ*g/ml of LPS. These results suggest that LPS induces NLRP3 and inflammatory gene expression in cultured macrophages.

### 3.3. E2 Inhibits LPS-Induced Macrophage Inflammation by Downregulating NLRP3

**Figure 3:**
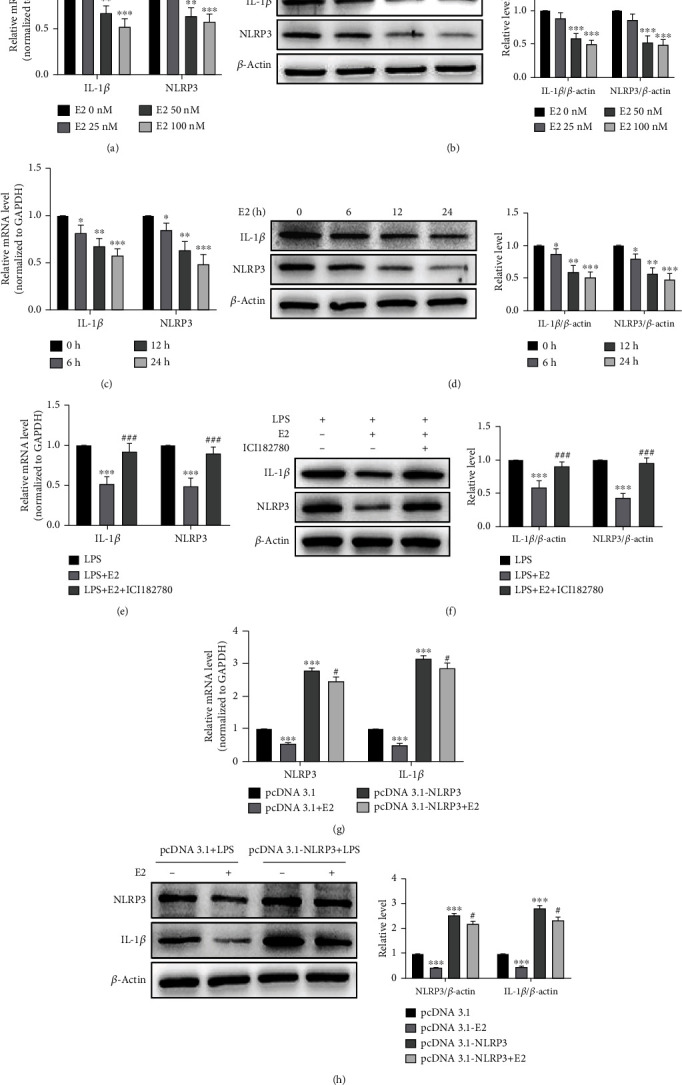
E2 inhibits LPS-induced macrophage inflammation by downregulating NLRP3. (a, b) RAW 264.7 cells were cultured in a medium containing 1 *μ*g/ml LPS and treated with different doses of E2 for 24 h. qRT-PCR (a) and Western blotting (b) detected the mRNA and protein expression of IL-1*β* and NLRP3. Relative expression of IL-1*β* and NLRP3 mRNA was presented after normalizing to GAPDH (mean ± SEM; *n* = 3). ^∗∗^*P* < 0.01 and ^∗∗∗^*P* < 0.001 vs. the E2 0 nM group, respectively. (c, d) RAW 264.7 cells were treated with 1 *μ*g/ml LPS and 100 nM E2 for different times. The expression of IL-1*β* and NLRP3 mRNA and protein was analyzed by qRT-PCR (c) and Western blotting (d). Relative expression of IL-1*β* and NLRP3 mRNA was presented after normalizing to GAPDH (mean ± SEM; *n* = 3). ^∗^*P* < 0.05, ^∗∗^*P* < 0.01, and ^∗∗∗^*P* < 0.001 vs. the 0 h group, respectively. (e, f) RAW 264.7 cells were treated with LPS (1 *μ*g/ml), LPS plus E2 (100 nM), or LPS plus E2 and ER antagonist ICI182780 (1 *μ*M). The expression of IL-1*β* and NLRP3 was analyzed and presented as in (a, b). Relative expression of IL-1*β* and NLRP3 mRNA was presented after normalizing to GAPDH (mean ± SEM; *n* = 3). ^∗∗∗^*P* < 0.001 vs. the LPS group. ^###^*P* < 0.001 vs. the LPS+E2 group. (g, h) RAW 264.7 cells were infected with pcDNA 3.1 or pcDNA 3.1-NLRP3 for 24 h, then incubated with 1 *μ*g/ml LPS and 100 nM E2 for 24 h. The expression of NLRP3 and IL-1*β* mRNA and protein was analyzed and presented as in (a, b). Relative expression of NLRP3 and IL-1*β* mRNA was presented after normalizing to GAPDH (mean ± SEM; *n* = 3). ^∗∗∗^*P* < 0.001 vs. the pcDNA 3.1 group. ^#^*P* < 0.05 vs. the pcDNA 3.1-NLRP3 group.

Because it is known that E2, the main endogenous estrogen, exerts multiple anti-inflammatory effects through the binding and activation of the intracellular ER*α* and ER*β* [26], we sought to know whether anti-inflammatory effects of E2 are related to its regulation of NLRP3. As shown by qRT-PCR and Western blotting, the addition of E2 to the LPS-treated macrophage led to dose-dependent decreases in mRNA and protein levels of NLRP3 and IL-1*β*, with their mRNA levels being reduced to less than 50% of the control upon exposure to 100 nM E2 (Figures 3(a) and 3(b)). Subsequently, we show that E2 decreases the level of NLRP3 and IL-1*β* mRNA and protein in a time-dependent manner, with a maximum decrease at 24 h after E2 exposure (Figures 3(c) and 3(d)). Pharmacological inhibition of ER*α* receptor activity by the ER*α* receptor antagonist ICI182780 completely abrogated inhibitory effects of E2 treatment on the upregulation of NLRP3 and IL-1*β* expression induced by LPS (Figures 3(e) and 3(f)), indicating that the inhibitory effect of E2 on these gene expressions is mediated through activation of estrogen receptors. In order to clarify whether E2 inhibits LPS-induced macrophage inflammation via NLRP3, we overexpressed NLRP3 by transfecting RAW 264.7 cells with pcDNA 3.1-NLRP3 and then treated the cells in LPS with or without E2. qRT-PCR and Western blotting analysis showed that treatment with E2 obviously decreased LPS-induced IL-1*β* expression in pcDNA 3.1-transfected cells, whereas the inhibitory effect of E2 was attenuated in pcDNA 3.1-NLRP3-transfected cells (Figures 3(g) and 3(h)), indicating that E2 inhibits LPS-induced macrophage inflammation partly by downregulating NLRP3. Altogether, these findings suggest that E2 inhibits LPS-induced macrophage inflammation through downregulating NLRP3.

### 3.4. miR-29a-5p Targets NLRP3

**Figure 4:**
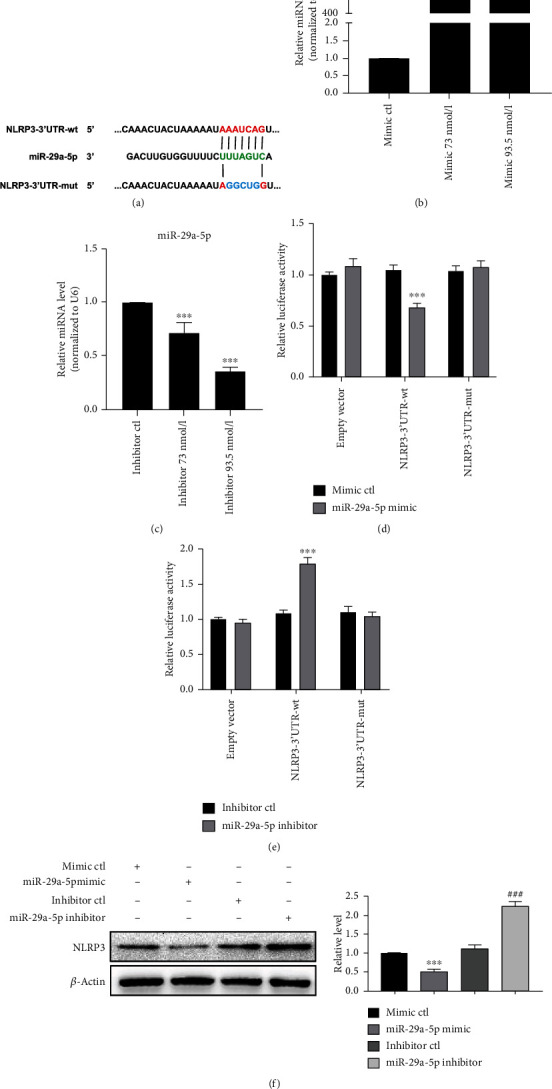
miR-29a-5p targets NLRP3. (a) Prediction of the miR-29a-5p binding site at NLRP3 3′-UTR. The red color indicates the sequence of the mutant miR-29a-5p binding site. (b) RAW 264.7 cells were transfected with the different concentrations of the miR-29a-5p mimic or mimic-ctl, and the miR-29a-5p level was detected by qRT-PCR. Relative expression of miR-29a-5p was presented after normalizing to U6 (mean ± SEM; *n* = 3). ^∗∗∗^*P* < 0.001 vs. the mimic-ctl group. (c) RAW 264.7 cells were transfected with the different concentrations of the miR-29a-5p inhibitor or inhibitor-ctl. qRT-PCR evaluated the expression of miR-29a-5p. Relative expression of miR-29a-5p was presented after normalizing to U6 (mean ± SEM; *n* = 3). ^∗∗∗^*P* < 0.001 vs. the inhibitor-ctl group. (d) Luciferase reporter assays were performed in 293A cells cotransfected cells with the miR-29a-5p mimic and wt or mutant (mut) NLRP3 3′-UTR-luciferase reporter. ^∗∗∗^*P* < 0.001 vs. the mimic-ctl group. (e) Luciferase reporter assays in 293A cells transfected with the constructs containing the wt or mutant NLRP3 3′-UTR after treatment with the inhibitor-ctl or miR-29a-5p inhibitor. ^∗∗∗^*P* < 0.001 vs. the inhibitor-ctl group. (f) RAW 264.7 cells were transfected with indicated constructs for 24 h; NLRP3 expression was detected by Western blotting. Left panel: representative blot from three independent experiments. Right panel: quantitation of NLRP3 normalized to *β*-actin. ^∗∗∗^*P* < 0.001 vs. the mimic-ctl group. ^###^*P* < 0.001 vs. the inhibitor-ctl group.

The above findings raise an important question about how NLRP3 expression is upregulated in LPS-treated macrophages. Because it is well known that the expression of many genes is regulated by miRNAs at the posttranscriptional level, we identified potential miRNAs targeting 3′-UTR of NLRP3 by using two target prediction programs, miRanda and TargetScan, and found 10 putative NLRP3-targeting miRNAs. Among them, only miR-29a-5p expression was significantly upregulated by E2 in LPS-treated RAW 264.7 cells (Figure S2). Next, computer-based sequence analysis (TargetScan and miRanda) was used to search for the potential matching site of miR-29a-5p in NLRP3 3′-UTR. As shown in Figure 4(a), there exists a putative miR-29a-5p binding site in NLRP3 3′-UTR. Because NLRP3 is a predicted target of miR-29a-5p, we first determined whether miR-29a-5p affected NLRP3 expression. To do this, RAW 264.7 cells were transfected with the miR-29a-5p mimic or its inhibitor, and transfection efficiency was detected by qRT-PCR. The results showed that the miR-29a-5p mimic dramatically increased, whereas its inhibitor markedly decreased the miR-29a-5p expression (Figures 4(b) and 4(c)). Then, 293A cells were cotransfected with the miR-29a-5p mimic and NLRP3 3′-UTR-luciferase reporter containing the wild-type (wt) or mutant (mut) miR-29a-5p binding site. The dual-luciferase reporter assay confirmed that transfecting 293A cells with the miR-29a-5p mimic significantly decreased the luciferase activity driven by wt-NLRP3 3′-UTR. However, mutation of the miR-29a-5p binding site in NLRP3 3′-UTR abolished the inhibitory effect of miR-29a-5p on luciferase activity (Figure 4(d)). Conversely, the cells cotransfected with the miR-29a-5p inhibitor significantly increased the luciferase activity driven by wt-NLRP3 3′-UTR, whereas mutation of the miR-29a-5p binding site in NLRP3 3′-UTR abolished the enhancement of luciferase activity (Figure 4(e)). We further transfected the miR-29a-5p mimic or inhibitor in RAW 264.7 cells and detected NLRP3 by using Western blotting. As shown by Western blotting, transfection of the miR-29a-5p mimic significantly reduced the NLRP3 level, while silencing of miR-29a-5p by its specific inhibitor increased the NLRP3 protein level (Figure 4(f)). Taken together, these results indicate that miR-29a-5p negatively regulates NLRP3 expression by directly targeting its 3′-UTR.

### 3.5. miR-29a-5p Reduces Inflammation Response in RAW 264.7 Cells Induced by LPS

**Figure 5:**
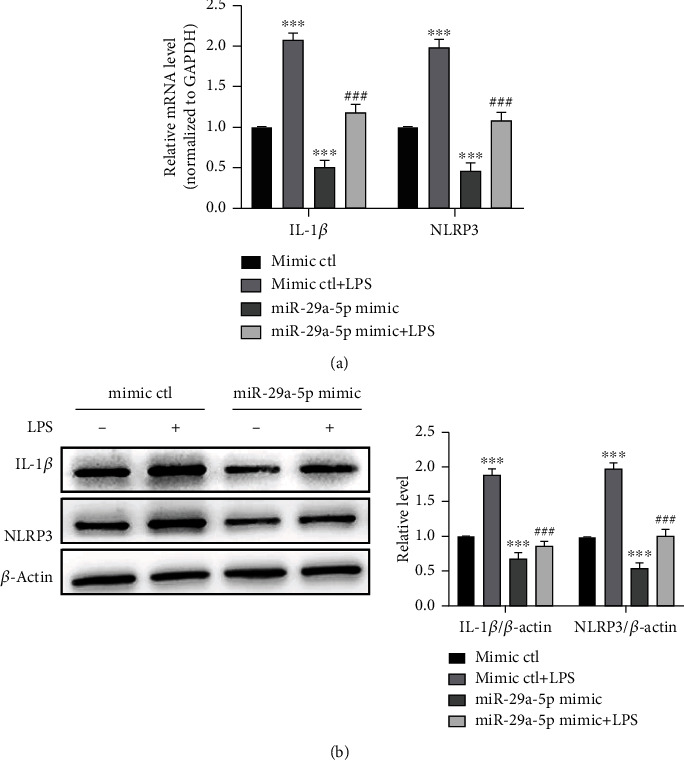
miR-29a-5p reduces inflammation response in RAW 264.7 cells induced by LPS. (a) RAW 264.7 cells were transfected with the miR-29a-5p mimic or mimic-ctl with or without LPS (1 *μ*g/ml) for 24 h. qRT-PCR was applied to detect mRNA expression of IL-1*β* and NLRP3. Relative expression of IL-1*β* and NLRP3 mRNA was presented after normalizing to GAPDH (mean ± SEM; *n* = 3). ^∗∗∗^*P* < 0.001 vs. the mimic-ctl group. ^###^*P* < 0.001 vs. the mimic-ctl+LPS group. (b) Western blotting detected IL-1*β* and NLRP3 expression in RAW 264.7 cells treated in (a). Left panel: representative blot from three independent experiments. Right panel: quantitation of IL-1*β* and NLRP3 normalized to *β*-actin. ^∗∗∗^*P* < 0.001 vs. the mimic-ctl group. ^###^*P* < 0.001 vs. the mimic-ctl+LPS group.

In order to further investigate the role of miR-29a-5p in macrophage inflammation, we transfected RAW 264.7 cells with the miR-29a-5p mimic or mimic-ctl and examined the effects of the miR-29a-5p mimic on NLRP3 expression as well as macrophage inflammation. As shown in Figures 5(a) and 5(b), both the mRNA and protein levels of NLRP3 and IL-1*β* revealed a <50%-fold decrease at 24 h after miR-29a-5p mimic transfection with or without LPS. These results strongly indicated that the upregulation of miR-29a-5p repressed expression of NLRP3 which is closely associated with macrophage inflammation.

### 3.6. Depletion of miR-29a-5p Promotes LPS-Induced Inflammation Response

**Figure 6:**
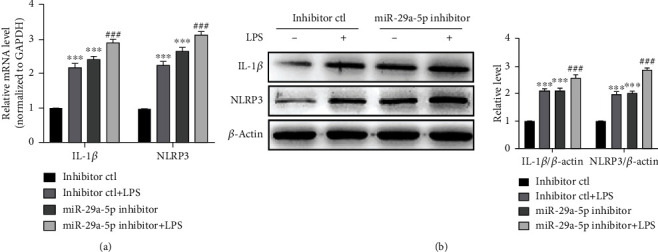
Depletion of miR-29a-5p promotes LPS-induced inflammation response. (a) qRT-PCR detected the mRNA expression of IL-1*β* and NLRP3 in RAW 264.7 cells transfected with the miR-29a-5p inhibitor or inhibitor-ctl and then treated with or without LPS (1 *μ*g/ml) for 24 h. Relative expression of IL-1*β* and NLRP3 mRNA was presented after normalizing to GAPDH (mean ± SEM; *n* = 3). ^∗∗∗^*P* < 0.001 vs. the inhibitor-ctl group. ^###^*P* < 0.001 vs. the inhibitor-ctl+LPS group. (b) Western blotting detected IL-1*β* and NLRP3 expression in RAW 264.7 cells transfected with the miR-29a-5p inhibitor or inhibitor-ctl and then treated with or without LPS (1 *μ*g/ml) for 24 h. Left panel: representative blot from three independent experiments. Right panel: quantitation of IL-1*β* and NLRP3 normalized to *β*-actin. ^∗∗∗^*P* < 0.001 vs. the inhibitor-ctl group. ^###^*P* < 0.001 vs. the inhibitor-ctl+LPS group.

To further identify the role of miR-29a-5p in macrophage inflammation, we knocked down the endogenous miR-29a-5p with an inhibitor of miR-29a-5p and examined the effects of the miR-29a-5p inhibitor on NLRP3 and IL-1*β* expression at mRNA and protein levels. We found that the transfected inhibitor-ctl (negative control) plus LPS treatment upregulated the expression of NLRP3 and IL-1*β* at mRNA and protein levels. Furthermore, when miR-29a-5p was knocked down by transfecting RAW 264.7 cells with the miR-29a-5p inhibitor, LPS-induced upregulation of NLRP3 and IL-1*β* became heavier (Figures 6(a) and 6(b)). Collectively, these data suggested that miR-29a-5p plays an important role in regulating inflammation gene expression of macrophages.

### 3.7. E2 Alleviates the Inhibition of miR-29a-5p Induced by LPS

**Figure 7:**
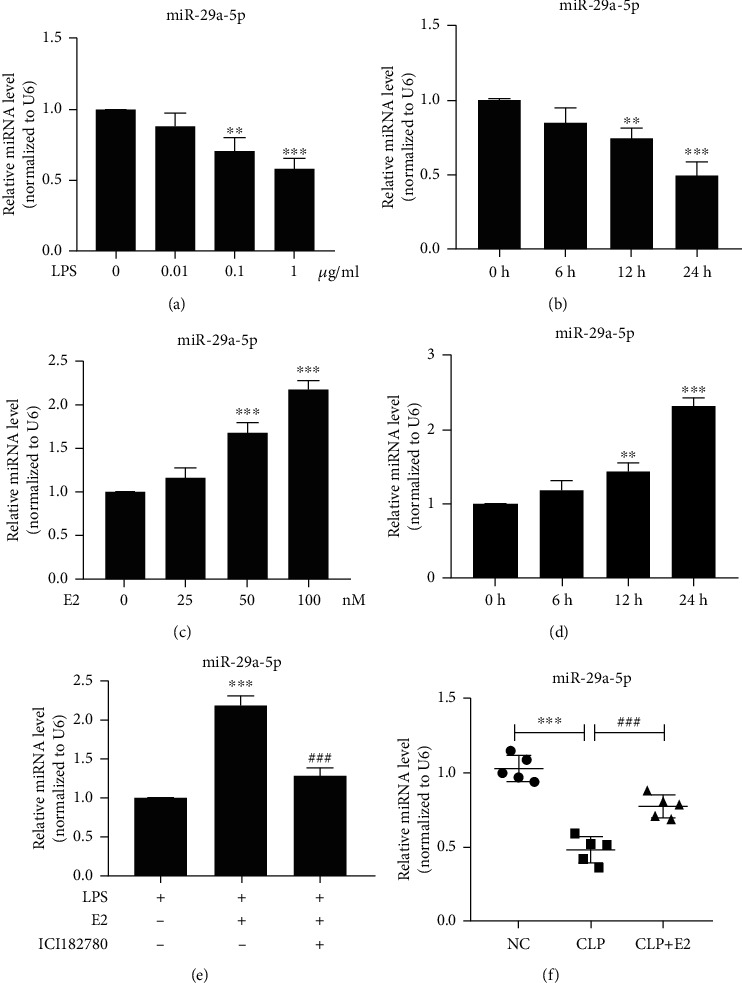
E2 alleviates the inhibition of miR-29a-5p induced by LPS. (a, b) RAW 264.7 cells were treated with the indicated concentrations of LPS for 24 h. qRT-PCR detected the expression of miR-29a-5p. Relative expression of miR-29a-5p was presented after normalizing to U6 (mean ± SEM; *n* = 3). ^∗∗^*P* < 0.01 and ^∗∗∗^*P* < 0.001 vs. the LPS 0 *μ*g/ml group, respectively. (b) RAW 264.7 cells were treated with 1 *μ*g/ml LPS for 0 h, 6 h, 12 h, and 24 h. The expression of miR-29a-5p was analyzed by qRT-PCR. Relative expression of miR-29a-5p was presented after normalizing to U6 (mean ± SEM; *n* = 3). ^∗∗^*P* < 0.01 and ^∗∗∗^*P* < 0.001 vs. the 0 h group, respectively. (c) RAW 264.7 cells were cultured in a medium containing 1 *μ*g/ml LPS and treated with different doses of E2 for 24 h. qRT-PCR detected the expression of miR-29a-5p. Relative expression of miR-29a-5p was presented after normalizing to U6 (mean ± SEM; *n* = 3). ^∗∗∗^*P* < 0.001 vs. the E2 0 nM group. (d) RAW 264.7 cells were cultured in a medium containing 1 *μ*g/ml LPS plus 100 nM E2 for the indicated times. qRT-PCR detected the expression of miR-29a-5p. Relative expression of miR-29a-5p was presented after normalizing to U6 (mean ± SEM; *n* = 3). ^∗∗^*P* < 0.01 and ^∗∗∗^*P* < 0.001 vs. the 0 h group, respectively. (e) RAW 264.7 cells were treated with LPS (1 *μ*g/ml), LPS plus E2 (100 nM), or LPS plus E2 and ER antagonist ICI182780 (1 *μ*M). The expression of miR-29a-5p was detected by qRT-PCR. Relative expression of miR-29a-5p was presented after normalizing to U6 (mean ± SEM; *n* = 3). ^∗∗∗^*P* < 0.001 vs. the LPS group. ^###^*P* < 0.001 vs. the LPS+E2 group. (f) qRT-PCR analysis of miR-29a-5p expression in NC-, CLP-, and CLP+E2-treated vascular tissues. Relative expression of miR-29a-5p was presented after normalizing to U6. *n* = 5 in each group. ^∗∗∗^*P* < 0.001 vs. the NC group. ^###^*P* < 0.001 vs. the CLP group.

Because it is known that LPS stimulates macrophage inflammatory response and that miR-29a-5p is involved in inflammatory response, we sought to determine whether the alteration of the miR-29a-5p expression profile is involved in LPS-induced macrophage inflammatory response. As shown in Figure 7(a), we showed that the presence of LPS in a concentration range from 0 to 1 *μ*g/ml decreased miR-29a-5p expression. Simultaneously, exposure of RAW 264.7 cells to LPS (1 *μ*g/ml) time-dependently depressed miRNA expression of miR-29a-5p, with the expression being significantly decreased at 24 h after LPS treatment and persisting to 24 h (Figure 7(b)). In addition, when the macrophage was incubated with LPS (1 *μ*g/ml) plus different doses of E2 for 24 h, miR-29a-5p expression was significantly upregulated, with their miRNA levels being increased to more than 2-fold of the control upon exposure to 100 nM E2 (Figure 7(c)). Parallel to that, when the macrophage was incubated with LPS (1 *μ*g/ml) plus 100 nM E2 for different times, miR-29a-5p expression was significantly upregulated (Figure 7(d)). The ER*α* receptor antagonist ICI182780 abrogated promotional effects of E2 treatment on the downregulation of miR-29a-5p expression induced by LPS (Figure 7(e)). We further tested miR-29a-5p expression in vivo. qRT-PCR results revealed that, compared with the NC group, the expression level of miR-29a-5p significantly decreased in the vascular tissues of the CLP group, whereas treatment with E2 obviously increased miR-29a-5p expression compared with the CLP group (Figure 7(f)). Altogether, these results suggest that E2 alleviates the inhibition of miR-29a-5p induced by LPS or CLP.

## 4. Discussion

Sepsis is a systemic inflammatory response syndrome caused by releasing various pathogenic microorganisms and endotoxin. Although the early prevention and treatment of sepsis have been widely spread and implemented, the morbidity and mortality of sepsis still remain high [27–29]. There is bacterial infection in sepsis. Bacteria can stimulate monocytes, macrophages, and neutrophils by secreting endotoxin, exotoxin, or LPS to induce immune response and inflammation [30]. Macrophage-mediated inflammation releases cytokines such as TNF-*α*, IL-1*β*, IL-6, and IL-8, which play an important role in the pathogenesis of sepsis. Therefore, the intervention of macrophage inflammatory response is an effective treatment strategy to inhibit the occurrence and development of sepsis. This study is aimed at elucidating the molecular mechanism of E2 inhibiting macrophage inflammatory response during sepsis. The main findings of this study are as follows. (1) E2 alleviates vascular inflammation in sepsis mice induced by CLP. (2) E2 can significantly reduce the inflammatory response of macrophages by downregulating the expression of NLRP3. (3) miR-29a-5p was significantly downregulated in LPS-treated macrophages. (4) E2 significantly upregulated the expression of miR-29a-5p in LPS-induced macrophages. (5) miR-29a-5p inhibits NLRP3 expression through directly targeting NLRP3 3′-UTR. Taken together, our study revealed that E2 exerts a significant anti-inflammatory efficacy by regulating the miR-29a-5p/NLRP3 axis in macrophages.

Although there are some controversies about the gender-specific response in patients with sepsis, it has been pointed out that gender plays an important role in the process of sepsis after injury. Clinical studies show that women have a significant advantage in morbidity and mortality of sepsis and septic shock [31–33]. The same results were obtained in animal experiments. Sepsis morbidity and mortality are higher in animals of male rodents, ovariectomized female rodents, or female rodents treated with testosterone [34, 35]. Interestingly, women of childbearing age with severe sepsis and shock are more tolerant of infection than menopausal women [36, 37]. Based on these phenomena, the increase of female hormone, especially the E2 level, is one of the important reasons for the improvement of antisepsis ability. The beneficial effect of estrogen under adverse circulatory conditions may be due to its positive effects on cytokine release, neutrophil chemotaxis, heat shock protein expression, HO-1 induction, and organ function recovery after shock and sepsis. Although there are many mechanisms for E2 to improve the antisepsis ability, there is still a lack of research on whether and how miRNA plays a role in this process. Our study demonstrated that E2 alleviates vascular inflammation in sepsis mice induced by CLP. In addition, further experiments suggested that E2 significantly reduced LPS-induced macrophage inflammation through miR-29a-5p.

MicroRNA acts as a posttranscriptional regulator in the pathogenesis of sepsis. Previous studies have shown that gracillin attenuates myocardial injury in sepsis mice by improving miR-29a and inhibiting LPS-induced apoptosis and inflammation [13]. lncRNA-MIAT is involved in sepsis-related renal injury by regulating the expression of miR-29a [38]. These results suggest that miR-29a plays an important role in sepsis. miR-29a-5p and NLRP3 can regulate inflammatory diseases together. miR-29a in astrocyte-derived extracellular vesicles inhibits cerebral ischemia-reperfusion injury through TP53INP1 and NF-*κ*B/NLRP3 [39]. The expression level of NLRP3 in sepsis patients was significantly increased [40]. The NLRP3 inflammasome enlarges inflammatory response, triggers apoptosis of immune cells, and aggravates the progress of sepsis. It is found that resveratrol exerts a protective effect on sepsis-associated encephalopathy by inhibiting the NLRP3/IL-1*β* axis of microglia [41]. In our experiment, LPS-induced upregulation of NLRP3 promoted the inflammatory response of macrophages, while E2 or miR-29a-5p mimics induced downregulation of NLRP3 which reversed the proinflammatory effect of LPS on macrophages.

Next, we further confirmed whether the inhibitory effect of E2 on macrophage inflammatory response is related to its regulation of miR-29a-5p expression by knockdown and overexpression of miR-29a-5p. Transfection of miR-29a-5p mimics significantly inhibited macrophage inflammation, while miR-29a-5p deletion aggravated upregulation of LPS-induced macrophage proinflammatory factor expression. More importantly, miR-29a-5p can directly bind to 3′-UTR of NLRP3, which provides a direct basis for miR-29a-5p to inhibit the expression of NLRP3. Our study is the first to confirm that E2 inhibits the inflammatory response of macrophages by regulating the miR-29a-5p/NLRP3 axis.

The main limitation of our current study is that only loss- and gain-of-function experiments have been done in vitro, and it needs to be further confirmed in vivo. In addition, clinical studies should be done in the future.

## 5. Conclusions

Collectively, our results demonstrate that E2 can effectively inhibit LPS-induced macrophage inflammation in vitro and sepsis-induced vascular inflammation in vivo by upregulating miR-29a-5p expression. Therefore, miR-29a-5p is an important target to regulate the inflammatory response of macrophages in sepsis. Targeting miR-29a-5p may be a novel therapeutic strategy to suppress sepsis-induced vascular inflammation.

## Data Availability

The data used to support the findings of this study are available from the corresponding authors upon request.

## Supplementary Materials

Supplementary Materials
*Supplementary 1*. Figure S1: the concentration of IL-1*β* in plasma at different time points. Figure S2: expression levels of miRNAs. *Supplementary 2*. Table S2: primers for real-time PCR.
