# Supplementary material for: 17β-Estradiol Attenuates LPS-Induced Macrophage Inflammation In Vitro and Sepsis-Induced Vascular Inflammation In Vivo by Upregulating miR-29a-5p Expression
Source: Mediators Inflamm. 2021 Jun 9;2021:9921897. doi: 10.1155/2021/9921897 (PMC8211527; doi:10.1155/2021/9921897)
Supplement: Supplementary Materials — Supplementary 1. Figure S1: the concentration of IL-1β in plasma at different time points. Figure S2: expression levels of miRNAs. Supplementary 2. Table S2: primers for real-time PCR. [file 9921897.f1.doc]

**SUPPLEMENTAL MATERIAL**

**17β-estradiol attenuates LPS-induced macrophage inflammation in vitro and sepsis-induced vascular inflammation in vivo by upregulating miR-29a-5p expression**

Man-li Zhang^1^, Hui Chen^1^, Zhan Yang^2, 3^, Man-na Zhang^4^, Xia Wang^1^, Kun Zhao^1^, Xuan Li^1^, Nan Xiu^1^, Fei Tong^1^*, Ya-xuan Wang^2^*

1 Department of Critical Care Medicine, The Second Hospital of Hebei Medical University, 215 Heping West Road, Shijiazhuang, Hebei, 050000, China

2 Department of Urology, The Second Hospital of Hebei Medical University, 215 Heping West Road, Shijiazhuang, Hebei, 050000, China

3 Department of Talent and Academic Exchange Center, The Second Hospital of Hebei Medical University, 215 Heping West Road, Shijiazhuang, Hebei, 050000, China

4 Department of Clinical Laboratory, The Second Hospital of Hebei Medical University, 215 Heping West Road, Shijiazhuang, Hebei, 050000, China

*Correspondence should be addressed to Fei Tong; tongfei168@163.com, and Ya-xuan Wang; [wangyaxuan87@126.com](mailto:wangyaxuan87@126.com)

**Figure S1**

Figure S1: The concentration of IL-1β in plasma at different time points. Blood samples of mice were collected before surgery (0 h) and at 6, 12, and 24 h after surgery. IL-1β in plasma was measured by ELISA. n=5 in each group. ****P* < 0.001 vs. 0 h group, respectively.

**Figure S2**

Figure S2: Expression levels of miRNAs. RAW 264.7 cells were cultured in medium containing 1μg/ml LPS and treated with or without E2 (100 nM) for 24 hours. Expressions of 10 potential miRNAs regulating NLRP3 were determined by qRT-PCR. Normalized against an internal control U6 RNA. ****P* < 0.001 vs. LPS group, respectively.

**Supplemental Table I**

3'UTR of NLRP3 contain miR-29a-5p target site or its mutated sequences

| **Gene** | **Sequences (5’-3’)** |
| --- | --- |
| NLRP3-wt-F: | TCGAGGTTATTTATCCAAACTACTAAAAATAAATCAGTTTACACATTTAAAATG |
| NLRP3-wt-R: | TCGACATTTTAAATGTGTAAACTGATTTATTTTTAGTAGTTTGGATAAATAACC |
| NLRP3-mut-F: | TCGAGGTTATTTATCCAAACTACTAAAAATAGGCTGGTTTACACATTTAAAATG |
| NLRP3-mut-R: | TCGACATTTTAAATGTGTAAACCAGCCTATTTTTAGTAGTTTGGATAAATAACC |

**Supplemental Table II**

| **Primer** | **Forward** | **Reverse** |
| --- | --- | --- |
| GAPDH | AAGGTGAAGGTCGGAGTC | GATTTTGGAGGGATCTCG |
| IL-1β | CAACCAACAAGTGATATTCTCCATG | GATCCACACTCTCCAGCTGCA |
| NLRP3 | CTACGGCCGTCTACGTCTTC | CAAATTCCATCCGCAGCCAG |

Primers for real-time PCR
